# Comparative effects of Facebook and conventional media on body image dissatisfaction

**DOI:** 10.1186/s40337-015-0061-3

**Published:** 2015-07-02

**Authors:** Rachel Cohen, Alex Blaszczynski

**Affiliations:** School of Psychology, Faculty of Science, The University of Sydney, Camperdown NSW, 2006 Australia

**Keywords:** Body image dissatisfaction, Social media, Appearance comparison

## Abstract

**Background:**

Appearance comparison has consistently been shown to engender body image dissatisfaction. To date, most studies have demonstrated this relationship between appearance comparison and body image dissatisfaction in the context of conventional media images depicting the thin-ideal. Social comparison theory posits that people are more likely to compare themselves to similar others. Since social media forums such as Facebook involve one’s peers, the current study aimed to determine whether the relationship between appearance comparison and body image dissatisfaction would be stronger for those exposed to social media images, compared to conventional media images.

**Methods:**

A sample of 193 female first year university students were randomly allocated to view a series of either Facebook or conventional media thin-ideal images. Participants completed questionnaires assessing pre- and post- image exposure measures of thin-ideal internalisation, appearance comparison, self-esteem, Facebook use and eating disorder risk.

**Results:**

Type of exposure was not found to moderate the relationship between appearance comparison and changes in body image dissatisfaction. When analysed according to exposure type, appearance comparison only significantly predicted body image dissatisfaction change for those exposed to Facebook, but not conventional media. Facebook use was found to predict higher baseline body image dissatisfaction and was associated with higher eating disorder risk.

**Conclusions:**

The findings suggest the importance of extending the body image dissatisfaction literature by taking into account emerging social media formats. It is recommended that interventions for body image dissatisfaction and eating disorders consider appearance comparison processes elicited by thin-ideal content on social media forums, such as Facebook, in addition to conventional media.

## Background

Body image dissatisfaction (BID), that is, the negative evaluations of one’s physical body, shape and weight [1], has consistently been implicated in the aetiology and maintenance of eating disorders (EDs) [2–4]. High rates of BID are prevalent among young Australian women indicating a need to identify the processes contributing to, and perpetuating, BID [5, 6]. BID is postulated to result from the discrepancy between societal and personal standards of appearance [7]. Accordingly, the mechanism of appearance comparison (AC), involving a direct comparison between such societal and personal standards of appearance, has been implicated in the development of BID [8]. This relationship between AC and BID has typically and consistently been demonstrated in the context of conventional media images [9, 10], whereby women exposed to magazine or television images of ‘thin-ideal’ models and celebrities engage in appearance-related comparisons and experience greater BID [6, 11–18].

Recent research suggests that social networking sites (SNSs: Facebook and Instagram) are increasingly used by college-aged females as the preferred social resource over conventional media forms, for example magazines and television [19]. Moreover, a growing literature suggests that SNSs have addictive properties [20]. Social comparison theory postulates that individuals are more likely to engage in comparisons with similar (peer groups) rather than dissimilar (others) personal attributes [21]. Accordingly, the relationship between AC and BID should be enhanced in SNSs where portrayed females are perceived as real, age- and status-related, and thus more personally identified with in contrast to professional models in conventional media [22].

Studies of self-presentation within SNSs have consistently found that users strategically manipulate their profiles in accordance with societal ideals of attractiveness [23–25]. Women viewing images of professional models represented in conventional forms of media remain aware that these have been digitally enhanced [26] thereby reducing the likelihood of self- comparison and propensity for BID [27]. In contrast, SNS images of ‘real’ women are assumed to be digitally unaltered and, hence accepted as more accurate and personally relevant [28].

Facebook is a popular SNS [29], with more than a billion active users [30]. Fifty eight percent of users are women [31] with users spending around an average of 16 h accessing Facebook per month [30]. In Australia there are currently 11,489,580 Facebook users, with the largest age group being 25–34 year-olds, followed by 18–24 year-olds [31].

Social media, unlike conventional media, also provides a virtual forum for *fat talk,* conversational threads about one’s own and other’s eating and exercise habits, weight concerns and ideal body shapes [32], thus serving to intensify the influence of AC on BID [33]. Typical Facebook profiles contain strategically selected thin photos of peers coupled with complimentary comments on appearance; for example “you look so skinny and beautiful” [24]. One study [34] found that 70.2 % of profiles of American undergraduate students referenced exercise and 12.3 %, eating habits with comments like “just did my morning work-out, feeling great!”. Of 600 Facebook users aged 16 to 40, 50 % reported that Facebook content made them more body-conscious; 31 % feeling “sad” as a result of comparing photos of themselves to those of Facebook friends, and 44 % reported desiring the same body or weight as Facebook friends [35].

Similarly, another study presented participants with a mock Facebook profile-picture and status that expressed a desire to lose weight, followed by their friend’s replies either encouraging or discouraging this weight-loss [36]. Participants reported greater BID and lower psychological well-being after reading peer-posted thin-promoting compared to thin- discouraging messages [36].

Time spent on Facebook appears to be associated with BID and ED pathology [37], with the relationship between Facebook and EDs stronger compared to viewing ‘Barbie’ type models on television and magazines [37]. In addition, adolescent Facebook users also score significantly higher on all body image concern measures than non users [38]. Although the correlational nature of these studies precludes causal inferences, these findings provide strong evidence implicating Facebook use as an emerging risk factor for BID.

It has been shown that conventional media images of the thin-ideal leads to BID via AC. Given the current increasing popularity of social media in comparison to conventional media, it is important to investigate these formats. Of interest is whether this relationship between AC and BID is stronger for those exposed to social media compared to conventional media. It was hypothesised that, the relationship between appearance comparison and changes in body image dissatisfaction from pre to post-exposure will be stronger for those exposed to Facebook compared to conventional media. Additionally, it was hypothesised that higher Facebook use will predict higher baseline body image dissatisfaction.

## Methods

### Participants

Two hundred and twenty-nine first year university female psychology undergraduate students were recruited through an online advertisement. Of this sample, 22 participants were excluded from analysis because they failed the manipulation check (see below) and 14 participants because they did not complete the full set of questionnaires. This resulted in a final sample size of 193 participants (response rate = 84.1 %).

An a priori power analysis for a multiple linear regression, assuming a maximum of 6 predictors in the model with a medium effect size (ƒ2 = .15) [39], indicated a total sample of *N* = 146 is sufficient to detect a significant difference at *p* = 0.05 (actual power = .952) [40]. Thus the resulting sample of 193 participants was sufficient.

Participants received course credit for participating in the study. Ages ranged from 17 to 46 years (*M* = 19.32, *SD* = 3.47). Of the sample, *n* = 90 (47.4 %) participants were Caucasian, *n* = 68 (35.8 %) Asian, *n* = 7 (3.7 %) Middle Eastern, *n* = 1 (0.5 %) Aboriginal/Torres Strait Islander, and *n* = 1 (0.5 %) African and *n* = 23 (12.1 %) other. One hundred and eighty-five (95 %) participants reported having a Facebook account, 6 (3 %) did not have one, and 3 (1.5 %) failed to indicate. Participants spent an average 15.78 h (*SD* = 12.62, *Range* = 0– 59.5) accessing Facebook each week. Participants were randomly allocated to either the Facebook (*n* = 102) or conventional media (*n* = 91*)* stimuli condition using the Qualtrics software program’s randomization function.

### Design

The study used a 2 × (2) mixed design. The between-group factor was type of exposure, and within-group factor, pre- to post-exposure. The dependent variables were appearance comparison (AC) and body image dissatisfaction change (BID-change) calculated by subtracting pre-exposure from post-exposure BID. Covariates were baseline thin-ideal internalisation, self-esteem and BMI.

## Materials

### Experimental stimuli

#### Facebook stimuli

Facebook stimuli consisted of ten slides depicting five mock profile-pages. The profiles were designed to reflect the realistic experience of viewing actual Facebook profiles. There were two slides per profile: thin-ideal profile-pictures (depicting thin females, comparable to those in the conventional media images), status’, peer comments, ‘tagged’ pictures and adverts in the side-bar, and; a ‘zoomed-in’ profile or tagged picture inclusive of friends’ comments as displayed when clicking on the picture. The presentation of Facebook stimuli in this way was intended to simulate a more realistic experience of Facebook, and to reflect the interactive nature of SNSs compared to conventional media images.

Stimuli were selected based on the ratings of a convenience sample of ten independent female university students. In order to ensure that the images were reflective of both attractive and thin images, raters viewed a series of 20 Facebook images and indicated the extent to which these were attractive and reflecting a thin-ideal using a 5-point Likert scale ranging from 1 (not at all) to 5 (to an extreme extent). Ten images receiving the highest scores (M = 4.1, SD = 1.12) were selected for use.

To enhance the external validity of the study, peer comments were derived from those found on real Facebook profile-pages, and participants were able to access stimuli from home computers rather than a laboratory site. The images and comments related to the beach, exercise, glamour and/or diet. (*Note.* all Facebook names were fabricated and photos were obtained via public access to Facebook).

#### Conventional media stimuli

Conventional print media stimuli consisted of ten thin-ideal commercial images of models/celebrities with themes matched to those in the Facebook profiles (i.e., beach, exercise, glamour and/or diet). The images consisted of magazine covers or advertisements found in popular magazines and presented in their original format. The same selection criteria used for the Facebook images was applied to the series of 20 Media images; thin-ideal *M* = 4.3, *SD* = 1.16.

#### Manipulation check

A manipulation check was included to ensure that participants paid adequate attention to the experimental stimuli. Participants were asked to select two pictures that they viewed on the stimuli images out of four possible choices; two stimuli pictures, and two closely matched distractors. Only participants who selected the correct two pictures were included in the analyses.

### Measures

#### Demographics

Participants self reported age, weight, height, ideal weight and race/ethnicity. Self-reported height and weight were used to calculate BMI (weight in kilograms divided by the square of the height in metres).

#### Facebook use

Participants self reported the extent and type of their Facebook use including number of years spent using, hours per day, times per day and days per week. Extent of Facebook use scores were calculated by hours per access x access per day x days accessed per week.

#### Type of Facebook use

Participants were asked how often they engaged in various Facebook activities such as looking at profiles of others and reading comments posted by others on a 5-point Likert scale ranging from 1 (never) to 5 (always). This metric was used to test the external validity of the stimuli by comparing the extent of similarity between actual Facebook use with experimental stimuli. For this study, the scale showed good reliability (*Cronbach’s alpha* = .828).

#### Thin-ideal internalisation

The 7-item Pressure subscale, one of the four subscales of the Sociocultural Attitudes Toward Appearance Questionnaire–Version 3 (SATAQ-V3) [41], was used to assess perceived pressure from media to attain the thin-ideal on a 5-point Likert scale ranging from 1 (strongly disagree) to 5 (strongly agree). Items were summed to obtain a score of thin-ideal internalisation at pre-exposure, with a high score indicating high internalisation of the media’s thin-ideal. Previous work found that the Pressure subscale (*alpha* = .84) significantly predicted high BID in a sample of 401 female undergraduates [42]. The scale showed good reliability (*Cronbach’s alpha* = .83).

#### Self-esteem

Rosenberg’s Self-Esteem Scale [43] was used to measure baseline self-esteem. This 10-item scale requires participants to indicate their agreement with each statement on a 4-point Likert scale ranging from 1 (strongly disagree) to 4 (strongly agree). Resulting scores range from 0 to 30, with higher scores indicating higher self-esteem. A study reported good reliability (*alpha* = .88) among a sample of Australian students [6]. The scale showed good reliability (*Cronbach’s alpha* = .88).

#### Body image dissatisfaction

The Body Areas Satisfaction Scale (BASS), one of the five subscales of the Multidimensional Body-Self Relations Questionnaire-Appearance Scales (MBSRQ) [44], was used to assess current perceptions of one’s body image, asking participants to reflect on their level of satisfaction with nine aspects of their physical appearance, “as you feel *right now”*. For the purpose of the current research question looking at changes in state BID following brief exposure, this subscale was used to measure state BID at pre and post-exposure. Items were scored on a 5-point Likert scale ranging from 1 (very satisfied) to 5 (very dissatisfied), with high scores indicating high state BID. The subscales showed acceptable reliability (*Cronbach’s alpha* = .789).

The literature shows that baseline BID moderates the relationship between media exposure and subsequent BID [7, 45–47]. Therefore it was necessary to account for pre-exposure BID. Pre-exposure BID scores were subtracted from post-exposure BID scores to create a BID- change score. Negative BID-change scores indicate a decrease in BID from pre to post- exposure, whereas positive BID-change scores indicate an increase in BID from pre to post- exposure.

#### Appearance comparison

A 3-item Extent Thoughts Questionnaire [11] was used to measure the extent to which participants had certain thoughts relating to AC while looking at the experimental images. Items, scored on a 5-point Likert scale ranging from 1 (not at all) to 5 (to an extreme extent), were averaged and summed to obtain a composite measure of AC. Higher scores indicate having experienced AC to a greater extent during exposure to the images. The scale previously showed good reliability (*alpha* = .80) in a sample of female undergraduates [11]. For this study, the scale showed excellent reliability (*Cronbach’s alpha* = .94).

Two additional questions, scored on a 5-point Likert scale ranging from 1 (not at all) to 5 (to an extreme extent), were included to assess the relevance of the stimuli to the individual’s actual daily exposure. The first item read, “I personally identify with the persona as shown in the images/Facebook profiles” and higher scores indicated higher personal identification with the stimuli personas. The second item read, “The types of images [and comments] I saw [on the Facebook profiles] are similar to those I see every day [when I sign into my own Facebook]” with higher scores indicating greater similarity between the experimental stimuli and the individual’s external exposure to Facebook or conventional media images.

#### Eating disorder risk

The Eating Attitudes Test-26 (EAT-26) [48] was used to identify participants at high risk of ED. This 26-item scale was scored on a 6-point Likert scale ranging from 1 (always) to 6 (never). This scale was reported to have excellent reliability (*alpha* = .90) among anorexia nervosa patients [48] and has been used to identify eating disturbances in non-clinical samples [49]. Item scores were summed for an overall test score, ranging from 0 to 78. Using recommendations for non-clinical samples [49], those who receive a score of 20+ are considered high risk of having an ED. For this study, the scale showed good reliability (*Cronbach’s alpha* = .870).

### Procedure

The Human Research Ethics Committee of The University of Sydney approved the study. Participants were emailed the participant information statement and given a URL to log into the questionnaire using Qualtrics software. Clicking onto the questionnaire indicated consent to participate. On completion of the pre-exposure questionnaires (demographics, Facebook use, baseline thin-ideal internalisation, BID and self-esteem) a link randomly allocated participants to one of two conditions:

- 30-s exposure to each of the ten slides, pertaining to five mock Facebook profile- pages (total 5 min exposure time; *n* = 102*)*; or
- 30-s exposure to ten commercial images of models/celebrities (total 5 min exposure time; *n* = 91*)*.

After viewing the profiles/images, participants were requested to complete post-exposure questionnaires (manipulation check, state AC, post-BID and ED risk).

### Data analyses

Data was analysed using PASW (SPSS) 20.0 software package. A series of hierarchical multiple regressions tested the relationship between AC and BID-change, and whether the type of exposure moderated this relationship. Multiple regressions were used to determine the relationship between level of Facebook use and baseline BID.

## Results

Extent of Facebook use was positively correlated with pre-BID, EAT-26 scores and AC indicating that greater time spent on Facebook was associated with higher baseline BID, risk for EDs and engagement in AC following exposure to thin-ideal stimuli (see Table 1). Extent of external Facebook activity similar to the activity elicited in the Facebook stimuli was significantly positively correlated with pre-BID, EAT-26 scores, pre-thin ideal pressure and AC. These correlations indicated that greater engagement in Facebook activities, similar to those elicited by the experimental stimuli, was associated with higher baseline BID, risk for EDs, baseline thin-ideal internalisation and engagement in AC following exposure to thin-ideal stimuli.

**Table 1:** Correlation Matrix of Study Variables (n = 193)

	Age	BMI	EAT	Pre-BID	BID-change score	Pre-Self-esteem	Pre-Thin-ideal internalisation	AC	Extent of Facebook use	Facebook activity similar to study	Personally identify with stimuli	Stimuli similar to everyday exposure
Age	1	.184^*^	-.001	-.121	-.121	.222^**^	-.022	-.211^**^	-.134	.000	-.075	-.149^*^
BMI		1	.157^*^	.235^**^	.011	-.016	.189^**^	.047	.003	.080	-.118	-.034
EAT			1	.397^**^	.172^*^	-.295^**^	.450^**^	.494^**^	.166^*^	.180^*^	.215^**^	-.008
Pre-BID				1	.001	-.572^**^	.373^**^	.485^**^	.196^**^	.162^*^	.071	-.048
BID-change score					1	-.144^*^	.312^**^	.357^**^	.013	.091	.101	-.095
Pre-Self-esteem						1	-.302^**^	-.374^**^	-.069	-.051	-.114	.003
Pre-Thin-ideal internalisation							1	.543^**^	.120	.173^*^	.150^*^	-.131
AC								1	.173^*^	.229^**^	.327^**^	.042
Extent of Facebook use									1	.276^**^	.021	.108
Facebook activity similar to study										1	.051	.129
Personally identify with stimuli											1	.070
Stimuli similar to everyday exposure												1

*Note.* BMI, Body Mass Index; EAT, Eating Attitudes Test; Pre-BID, Pre-exposure Body Image Dissatisfaction; BID-change score, Body Image dissatisfaction change score; AC, Appearance Comparison

*. Correlation is significant at the 0.05 level (2-tailed)

**. Correlation is significant at the 0.01 level (2-tailed)

A positive BID-change score indicates an increase in BID, and a negative score, a decrease following exposure. Means and standard deviations for pre-BID, post-BID and BID-change scores in both groups are presented in Table 2.

**Table 2:** Descriptive Statistics for Pre-BID, Post-BID and BID-Change Scores in Both Exposure Groups

	Pre-BID		Post-BID		BID-change score (Post-Pre)	
	*M*	*SD*	*M*	*SD*	*M*	*SD*
Media (*n* = 91)	2.939	.664	3.062	.705	.123	.289
Facebook (*n* = 102)	2.960	.600	3.180	.677	.220	.274

A hierarchical multiple regression was conducted to test the hypotheses that type of exposure moderated the relationship between AC and BID-change. Since baseline self-esteem and thin- ideal internalization have been shown to moderate the effects of exposure on BID-change [7, 45–47], these two variables were entered on the first line as covariates, followed by type of exposure (dummy-coded: Media = 0, Facebook = 1), AC and their interaction term (with AC mean-centred). Despite the theoretical rationale for BMI [46], it did not significantly correlate with BID-change in the current study and therefore was not included in the analysis.

The full model predicting BID-change from pre-self-esteem, pre-thin-ideal internalisation, AC, exposure and the interaction between AC and exposure was significant and accounted for 15.7 % of the variance in BID-change, *R*^2^ = .157, *F (*5,187) = 6.970, *p* < .01; adjusted *R*^2^ = .135. The only significant predictor of BID-change was AC, indicating that, controlling for the other variables in the model, for every one point increase in AC, BID scores are expected to increase by .073 units following exposure (*B* = .073, *t* = −3.079, *p* = .002). None of the other variables, including the interaction term, independently predicted BID-change. Table 3 lists the hierarchical multiple regression results.

**Table 3:** Summary of the Hierarchical Regression Analysis Predicting BID-Change (n = 193)

Model	Variable	*B*	*SEB*	β	*R* ^2^	*R* ^2^ *change*	*P*
1	Intercept	-.081	.162				
	Pre-thin-ideal internalisation	.015	.004	.296			.000**
	Pre-self-esteem	-.030	.040	-.055			.451
					.100	.100	
2	Intercept	-.136	.178				
	Pre-thin-ideal internalisation	.007	.004	.136			.110
	Pre-self-esteem	-.005	.041	-.010			.895
	AC	.066	.020	.267			.001**
	Exposure	.054	.041	.096			.182
					.155	.055	
3	Intercept	-.139	.178				
	Pre-thin-ideal internalisation	.007	.004	.136			.108
	Pre-self-esteem	-.013	.043	-.023			.764
	AC	.073	.024	.298			.002**
	Exposure	.056	.041	.098			.172
	AC*exposure	-.022	.035	-.057			.540
					.157	.002	

*Note.* The dependent variable was BID-change; *B* = unstandardized regression coefficient; *SEB* = standard error of the coefficient; β = standardized coefficient; *p < .05, **p < .01

To test the relationship between AC and BID-change for each type of exposure, two separate hierarchical multiple regressions were run predicting BID-change from AC for Facebook exposure (*M* = −3.108, *SD* = 1.213) and media exposure (*M* = 2.810, *SD* = 1.079). In both analyses, thin-ideal internalisation and self-esteem were entered on the first step as covariates, followed by AC.

### Media exposure

For those exposed to media, the full model predicting BID-change from thin-ideal internalisation, self-esteem and AC was significant and accounted for 9.4 % of the variance in BID-change, *R*^*2*^ = .094, *F* (3,87) = 3.017, *p* = .034; adjusted *R*^*2*^ = .063. Adding AC did not significantly account for more variance in BID-change above that of thin-ideal internalisation and self-esteem and AC was not a significant predictor of BID-change for those exposed to media (*B* = .054, *t* = 1.440 *p* = .153).

### Facebook exposure

For those exposed to Facebook, the full model predicting BID-change from thin-ideal internalisation, self-esteem and AC was significant and accounted for 17.3 % of the variance in BID-change, *R*^*2*^ = .173, *F* (3,98) = 6.841, *p* < .01; adjusted *R*^*2*^ = .148. Adding AC significantly accounted for 8.1 % more variance in BID-change above that of thin-ideal internalisation and self-esteem *F* (1,98) = 9.566, *p* < .003. Controlling for thin-ideal internalisation and self-esteem, AC was a significant predictor of BID-change, indicating that for everyone one point increase in AC, BID scores were expected to increase by .074 points following exposure to Facebook stimuli (*B* = .074, *t* = 3.093, *p* = .003). Table 4 lists the hierarchical multiple regression results for each type of exposure.

**Table 4:** Comparison of the Hierarchical Regression Analyses Predicting BID-Change for Each Type of Exposure (Media and Facebook)

	Media Exposure (*n* = 91)								Facebook Exposure (*n* = 102)						
Model	Variable	*B*	*SEB*	β	*t*	*R* ^2^	*R* ^2^ *change*	*P*	*B*	*SEB*	β	*t*	*R* ^2^	*R* ^2^ *change*	*P*
	Intercept	−0.077	0.267						0.053	0.225					
1	Pre-thin-ideal internalisation	0.014	0.007	0.238	2.011			0.047*	0.014	0.005	0.273	2.803			0.006**
	Pre-self-esteem	−0.028	0.059	−0.056	−0.471			0.639	−0.056	0.059	−0.093	−0.952			0.344
						0.073	0.073						0.092	0.092	
2	Intercept	−0.251	0.291						−0.050	0.219					
	Pre-thin-ideal internalisation	0.009	0.007	0.161	1.25			0.215	0.006	0.005	0.116	1.086			0.280
	Pre-self-esteem	0.012	0.065	0.025	0.189			0.851	−0.034	0.057	−0.057	−0.607			0.545
	Appearance comparison	0.054	0.037	0.200	1.440			0.153	0.074	0.024	0.330	3.093			0.003**
						0.094	0.022						0.173	0.081	

*Note.* The dependent variable was BID-change; *B* = unstandardized regression coefficient; *SEB* = standard error of the coefficient; β = standardized coefficient

*p < .05, **p < .01

To test the prediction that the extent of Facebook use would predict baseline BID, a multiple regression analysis was run, controlling for baseline self-esteem, BMI and pre-thin-ideal internalisation (based on theoretical rationale [50] and the significant correlations between these variables and baseline BID). The overall model was significant, accounting for 40.3 % of the variance in baseline BID *R*^2^ = .403, *F (*4,177) = 29.880, *p* < .01; adjusted *R*^2^ = .390. Controlling for the other predictors in the model, extent of Facebook use was a significant predictor of baseline BID, such that for every one hour increase in Facebook use, BID is expected to increase by .007 points (*B* = .007, *t* = −2.327, *p* = .021). Table 5 lists the multiple regression results.

**Table 5:** Summary of the Multiple Regression Analysis Predicting Baseline BID (n = 193)

Variable	*B*	*SEB*	β	*R* ^2^	*P*
Intercept	3.473	.337			
Pre-self-esteem	-.608	.075	-.487		.000**
Pre-thin-ideal internalisation	.021	.007	.182		.004**
BMI	.029	.009	.195		.001**
Extent of Facebook use	.007	.003	.136		.021*
				.403	

*Note.* The dependent variable was baseline BID; *B* = unstandardized regression coefficient

*SEB* = standard error of the coefficient; β = standardized coefficient; *p < .05, **p < .01

To determine the degree of perceived similarity between experimental stimuli and Facebook/media images, responses to the question, “*the types of images I saw in the stimuli were similar to what I see everyday*” were analysed. On average participants exposed to media stimuli found the stimuli significantly more similar to what they reported seeing every day (*M* = 3.32, *SD* = 1.191) compared to those exposed to the Facebook stimuli (*M* = 1.81, *SD* = 1.069), *t* (1,191) = 9.249, *p* < .001.

The extent of Facebook use and ED risk was assessed. Participants scoring higher than or equal to 20 on the EAT-26 were considered at high risk, and those less than 20, at low risk for an ED [49]. The average extent of Facebook use was 21.54 h per week (*SD* = 16.715) for those in the high-risk group and 14.91 h per week (*SD* = 11.606) for those in the low- risk group.

Extent of Facebook use was not normally distributed across risk groups. A Mann–Whitney *U* test was therefore run to determine differences in extent of Facebook use between those at high- (EAT-26 score > =20, *n* = 26) compared to low-risk (EAT-26 score <20, *n* = 157) for an ED.

Facebook use scores for high risk (mean rank = 108.04) were statistically significantly higher than for low risk (mean rank = 89.34), *U* = 1624, z = −1.669, *p* = .045.

## Discussion

To the best of the authors’ knowledge, this is the first study to compare Facebook and conventional media in their effects on BID using an experimental design. It was hypothesised that the relationship between AC and BID-change would be stronger for those exposed to Facebook images compared to those exposed to conventional images. Although AC was a significant predictor of BID-change for those exposed to Facebook, and not for those exposed to conventional media, type of exposure did not moderate this relationship. In other words, there was no indication of significant differences between Facebook and conventional media in their effects on the relationship between AC and BID-change. Although unexpected, there are a number of possible explanations why a moderating effect was not obtained.

The relationship between AC and BID is said to occur when one is exposed to thin-ideal content [51, 52]. In the current study, both stimuli represented thin-ideal content. Accordingly, the non-significant moderating role of type of exposure may be due to ceiling effects. The high degree of thin-ideal content in both forms of stimuli may have led both groups to experience high amounts of AC and BID, thus limiting the capacity for differences to be found between the two exposures. Previous studies investigating the effects of thin-ideal exposure on BID compared contrasting stimuli, for example over-weight females versus thin females [53–55], thin-ideal stimuli versus neutral stimuli [17] and attractive females versus objects [7]. Such dissimilar stimuli may facilitate the detection of significant differences; however, these were not deemed to be appropriate for the current study that specifically aimed to delineate the differences between thin-ideal content depicted in conventional and social media. The trends found in the current study indicate that there might be an additive effect of the social component of Facebook on AC.

The finding that exposure did not moderate the relationship between AC and BID-change was also unexpected in light of the assertion that females tend to compare themselves more with similar and self-relevant others [21]. One possible explanation is that participants may have been more familiar with celebrity models depicted in the conventional media stimuli, and hence perceived as more relevant targets of comparison compared to Facebook stimuli, who were completely unknown to the participants [22, 56]. In response to the statement, “*the types of images I saw in the stimuli were similar to what I see everyday”,* participants exposed to the conventional media indicated that the images in the study were more similar to what they see every day compared to those in the Facebook group. Moreover, females in the Facebook images were selected because they represented the thin-ideal and thus did not look vastly different from the models in the media stimuli. Consequently, the participants may have perceived the Facebook ‘friends’ as models, similar to those in the conventional media images.

It has been suggested that Facebook friends, who are usually highly similar and socially relevant to the Facebook user, may elicit more significant comparative processes [57]. Given the media stimuli were perceived as more relevant and familiar to participants in the current study, the non-significant differences between the two groups may indicate that the experimental stimuli were not able to elicit the social nuances distinctive to Facebook profiles. Thus, future studies comparing the effects of Facebook and conventional media images on AC and BID should aim to use Facebook profiles pertaining to the participants’ actual peers.

Finally, the non-significant differences may indicate that Facebook and conventional media may be comparable in their effects on AC and BID. This has implications both theoretically and practically, given that thin-ideal images in conventional media have consistently been found to have detrimental effects on BID and broader mental health issues [7, 16, 47, 58–60]. Moreover, there is increasing evidence of the high user rates of Facebook and other SNSs over that of conventional media [19]. The present results indicate that Facebook, like conventional media, may play a similar role in the development of BID.

The hypothesis that higher Facebook use is associated with higher baseline BID was supported. Furthermore, results indicated that the extent of Facebook use accounted for unique variance in baseline BID above that of self-esteem, BMI and thin-ideal internalisation, variables already established as risk factors for BID [46]. This finding suggests that high Facebook use may be considered a risk factor for BID, even when controlling for self-esteem, BMI and thin-ideal internalisation.

Of interest was the considerably higher average Facebook use in the current sample compared to previous frequency studies. Whereas the average use in Australia is 15.55 h per month [31], the average use in the current sample was 15.78 h per week (i.e., greater than 60 h per month). This notably higher use may be due to the fact that the sample was comprised of only females, the dominant gender in Facebook use within the age group with the highest reported Facebook use [31]. In light of these demographics, the higher Facebook use in the current study may not be surprising. Nevertheless, caution should be taken when generalising the current findings to older samples or the general population.

An important finding was the relationship between Facebook use and ED risk. Post-hoc analyses revealed that Facebook use was significantly higher for those at high risk of EDs compared to those at low risk. This finding was consistent with a recent survey that found an association between time spent on Facebook and ED pathology [37]. The correlational finding could indicate that excessive Facebook use is associated with an individual’s risk of developing an ED. The integrated cognitive-behavioural theory of EDs [61] identifies a feedback loop whereby exposure to body-related stimuli activates and reinforces an over- concern with one’s own body, which in turn reactivates attentional biases toward body- related stimuli. The frequency of this feedback loop serves to generate or maintain EDs [61] and this process could possibly explain the finding of a relationship between frequent Facebook use and ED risk. Accordingly, it may be the case that frequent exposure to thin- ideal content on Facebook reinforces one’s own body-related concerns, eliciting cognitive biases that lead one to selectively attend to thin-ideal content on Facebook [37].

On the other hand, it may be possible that people with a higher risk of EDs are more likely to use Facebook, which may in turn serve to reinforce their ED risk [62]. Cognitive models of EDs posit that individuals with EDs show a selective attention for appearance-related cues [61]. Thus, the current study’s finding of an association between ED risk and high Facebook use could imply that people with EDs may be more vulnerable to the negative effects of Facebook. Either way, the current results highlight the need for further research into the relationship between ED risk and Facebook use. Moreover, this study operationalized ED risk using recommendations for a non-clinical sample [49]. Future research with clinically diagnosed ED samples may prove to be more useful in examining the role of Facebook use in the development and maintenance of EDs.

As in all studies there are limitations. All data collected was self-report, including self- reported weight and height, which is subject to recall errors or biased reporting. A main caveat of the current study is a lack of external validity for the Facebook stimuli, which contained mock personas rather than the participants’ actual peers. Therefore Facebook’s additional peer-relevant component was less salient. The current study attempted to increase external validity by designing stimuli that replicated real Facebook profile-pages, with participants able to access the experimental stimuli from their own computer at leisure.

Another potential limitation was that the current study only included female ‘friends’ without also including comments by male ‘friends’. The Catalyst Model [63] argues that BID is a direct outcome of competition between females for mates. In social contexts, women understand that they are competing for sexual partners with their peers, in contrast to models or celebrities where there is no perceived competition [64]. Furthermore, female AC tendencies have been found to be heightened in the context of potential male partners [65]. Given that Facebook friends typically consist of both females and males, and that Facebook is used to develop or maintain both social and romantic relationships, it may have been informative to include both male and female images and comments in the experimental stimuli. It is possible that these additions may have led to increased AC and thus greater BID than for those exposed to conventional media images, void of a social or romantic context.

It is also possible that the difference between conventional media and Facebook exposure was not evident in this short-term study, but may have a differential influence on BID in the long-term. It has been argued that whilst it may be difficult to capture the effects of a brief exposure to thin-ideal images in a laboratory setting, continuous exposure to thin-ideal media in everyday life may serve to reinforce BID in the long-term [10, 60]. The duration of the exposure to Facebook images in the current study was 5 min in total whereas the average amount of time per Facebook access is typically 20 min, numerous times a day [31]. Moreover, the trends in the current study showed that AC was greater for those exposed to Facebook than conventional media. Therefore despite the fact that there were no significant findings of an acute effect of exposure to Facebook on BID over conventional media in the present study, it is plausible that continued exposure to Facebook thin-ideal content could have dire effects in the long-term, above that of conventional media [60]. Longitudinal research is needed to further examine the long-term effects of such a relationship.

## Conclusion

This study’s findings have several theoretical and practical implications. The current study’s finding that the relationship between AC and BID was comparable between Facebook and conventional media, suggests that Facebook, and potentially other SNSs, are key factors that need to be considered in the development of BID.

Additionally, the finding that AC is affected by Facebook, not just conventional media, suggests that interventions should focus on how people compare themselves with peers in social media, in addition to how they view models in conventional media images. The finding of an association between the extent of Facebook use and ED risk indicates that, although thin-ideal forums such as Facebook do not cause EDs, they may be one of many maintaining factors, reinforcing an over-evaluation of weight and shape [61].

Although this explorative study failed to demonstrate that the relationship between AC and BID is stronger following exposure to Facebook than conventional media in the short-term, it suggested that exposure to Facebook is at least on par with conventional media exposure in its detrimental effects on BID via AC.

The present study contributes to the existing literature on the media’s influence on BID by expanding upon a narrow focus on conventional media only by exploring emerging social media formats.

## Abbreviations

BID: Body image dissatisfaction
BID-Change: Body image dissatisfaction change score
AC: Appearance comparison
ED: Eating disorder
BMI: Body mass index
SNS: Social networking site
